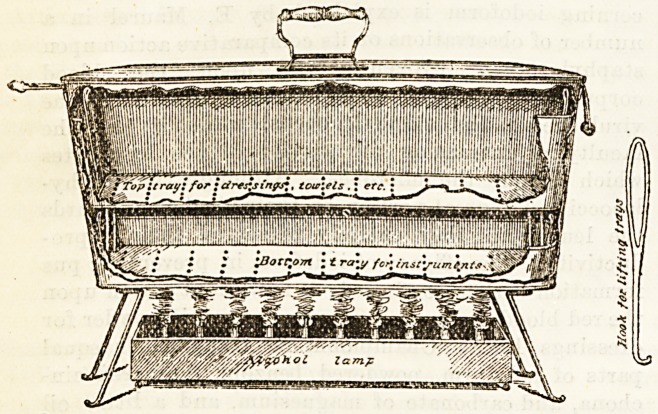# General Surgery

**Published:** 1894-07-07

**Authors:** 


### GENERAL SURGERY.

**Antiseptics.**—Notwithstanding the fact that many surgeons have almost entirely given up the use of chemical antiseptics for operations, it is astonishing to note the number of new discoveries that are constantly being made and added to the list of agents in other departments of surgery. Old drugs are discarded, and it becomes quite a difficult matter for practitioners to decide and depend upon any particular remedy. In this connection it is interesting to observe that the reviewer of the recent edition (1893) of John Ashhurst, jun.'s, excellent work, "The Principles and Practice of Surgery," calls[1] attention to the statement by the author "that while recognising many advantages in the methods of modern aseptic and antiseptic surgery, he has not found any marked diminution in mortality resulting from their use." In order to ascertain the best disinfectant for dental purposes, viz.: for diseased condition of the oral cavity, alveolar abscess, putrid pulp, &c., C. M. Stewart[2] has carried out a series of experiments showing the relative quickness of action of bichloride of mercury, oil of peppermint, permanganate of potash, chlorophenique, Listerine, saccharine, and some of the other products of coal tar distillation upon staphylococcus pyogenes aureus, and albus, the germs most often found in the mouth. Lysol more nearly answered the true definition of a dental disinfectant than any agent experimented with. Europhen has been used by Oefelein and Neuberger[3] in soft sores, balanitis, burns, phagedænic soft ulcers, suppurative lupus, &c. Great drying powers without local irritation and absence of smell are the principal advantages as compared with iodoform, and it proved most valuable in erosions and fissures. It adheres better than iodoform, and smaller quantities are required. Gilbert[4] recommends its use alternately as a powder and a 2 per cent. ointment for varicose and other ulcers. It has not any marked poisonous properties. The contradictory views held by biologists and clinicians concerning iodoform is explained by E. Maurel[5] in a number of observations on its comparative action upon staphylococcus pyogenes, and upon the blood corpuscles. He finds that iodoform attenuates the virulence of the staphylococcus, while it has the faculty of increasing the activity of the leucocytes which devour the microbes. Although the staphylococci lost a great portion of their virulence towards the leucocytes they entirely preserved their reproductivity. The efficacy of iodoform in preventing pus formation is thus confirmed; it is without action upon the red blood corpuscles. As an antiseptic powder for dressings Lucas Championnière[6] recommends equal parts of iodoform, powdered benzoin, powdered cinchona, and carbonate of magnesium, and a little oil of eucalyptus. In this mixture the odour of iodoform is said to be masked. C. Steinmetz[7] records his experience with oxychinaseptol or diaphtherin as an antiseptic in about forty cases. It is serviceable in a 1 per cent. solution for irrigation and moist dressings. It has a caustic action as a 10 to 50 per cent. ointment with vaseline. Although soluble in water, it has the disadvantage of staining instruments and colouring the nails yellow. Lysol is extolled by C. B. Adams[8] for its great solubility, its power in weak solutions, its slight poisonous qualities, cheapness, and great value as a deodoriser. E. P. Murdock[9] thinks it as efficient as bichloride, and because of its anæsthetic properties recommends it as a most satisfactory dressing for burns. Rudolf Abel[10], from careful experiments, concludes that the icthyol preparations in weak solutions in a short time destroy the pyogenic and erysipelas streptococci. Icthyol may therefore be used with success in the suppuration from these cocci. The diphtheria bacillus in fresh colonies is easily destroyed by weak icthyol solutions, while mature ones are acted upon with difficulty. Hence it is useful in diphtheria only in prophylaxis. Camphorated carbolic acid is recommended by Toms[11] as an antiseptic agent. A 50 per cent. combination does not irritate mucous surfaces. It retains the full antiseptic and germicidal properties of the acid, without its destructive action to living tissue. Toms used it diluted with 50 per cent. of cotton seed oil for a foul and extensive ulcerating epithelioma of the leg, and also for recently lacerated wounds. M. Lenti[12] has recently verified the opinion which Koch gave in 1881, that carbolic acid loses almost all its germicidal properties when dissolved in alcohol (in the absence of water), oil or glycerine. He finds the same with regard to sublimate and lysol and strongly insists that on account of their influence these dissolvents,—alcohol, glycerine, and fats—should be avoided in the preparation of disinfecting fluids. Berlioz[13] has devised an antiseptic varnish (steresol) for covering diseased skin and mucous membrane. It is a mixture of gum lac with benzoin and Tolu balsam, the whole being dissolved in alcohol and impregnated with carbolic acid 10 per cent. Essence of caneila will mask the disagreeable odour of the phenol, while saccharine will remove the bitter taste. The varnish can be applied by pencilling after drying the surface; it resists the action of the saliva when applied to the throat. C. B. Lockwood[14] reports upon the details of many of the ordinary methods of skin disinfection in surgical operations, and the failures resulting from their use. The bacteria of the skin reside, not only upon its surface, but also in the hair follicles, sebaceous glands, and sweat ducts. In twenty-one experiments upon the disinfection of the skin, seven were successful, the others became septic and showed the presence of bacteria. Of late he has used biniodide of mercury (1 in 4,000) to rinse the skin after washing and scrubbing with soft soap and water; a dressing of carbolic gauze soaked in glycerine of biniodide (1 in 4,000) is then applied. As to the disinfection of the hands, the nails should be cut as close as possible, and the hands scrubbed with hot water and soap (the nail-brush being sterilised for half an hour), and then soaked for a minute in a 1 in 1,000 solution of sublimate in rectified spirit. Lockwood's experiments upon disinfection of the skin of the patients and of the operator's hands seem to favour the assumption that glycerine and alcohol are better than water for the dilution of the chemicals. Towels should be opened out and steamed for half an hour in an ordinary steam steriliser, and afterwards soaked in an antiseptic. Sponges, well shaken to get rid of all sand, are left in a solution of hydrochloric acid (ʒi. to Oi) for twenty-four hours, to remove the bits of shell and coral, and thoroughly washed in warm water previously boiled. They are then placed for half an hour in a warm solution of washing soda (ʒi. to Oi) to remove fat and albumen (this process requires several repetitions if the sponges are full of blood, fat, and albumen), afterwards rinsed in warm sterilised water, and then immersed in cold solution of sulphurous acid (1 in 5) for 12 hours. Lastly, they are squeezed dry and placed in carbolic lotion (1 in 20) in well-stoppered jars. A handy portable steriliser for operative work in private practice has been constructed according to the well-known Koch's steampot by Willy Meyer,[15] of New York. It consists of a nickel-plated copper kettle, top 17 in. by 8½ in., bottom 15½ in. by 7 in., height 6½ in., with two lateral wooden handles and a lid. In its bottom is a perforated tray, as in Rotter's apparatus, with a brim 1½ in. high, and two metal handles for lifting out. A second tray, with deeper brim 2½ in., stands above this, on legs, for dressings, &c. An alcohol lamp, with ten Bunsen burners, and a stand of nickel-plated iron, complete the apparatus. Two smaller sizes are also made. Half-an-hour is needed for sterilisation, and all material, dressings, instruments, &c., which will come in direct contact with the wound and its immediate surroundings may be carried to the patient's house and sterilised just before the operation. The soda and water having been poured into the kettle, the instruments are put into the bottom tray twenty-five minutes after the dressings, &c., have been inserted in the top tray, as they require only five minutes' boiling. The apparatus is self-acting, and by the time (30 minutes) the lamp, with its requisite supply of alcohol, extinguishes, everything in the apparatus is sterilised and the gauge almost entirely dry.

H. Hochenegg[16] gives a simple, practical, and cheap method of stamping articles of dressing that have been sterilized. The articles are marked with date in certain parts by means of colouring matter, consisting of a mixture of 150 parts of solution of aluminum acetate (Austrian Pharmacopœia), 150 parts of fountain water, and 5 parts of 20 per cent. paste of alizarine. This becomes bright red on exposure to the temperature of boiling water. If the sterilization has been done effectively and satisfactorily all mistakes are avoided.

[1] American Journal of Med. Sciences, Feb., 1894. [2] The Pharmaceutical Journal, Dec. 30, 1893, p. 525. [3] Epit. B. M. Journal, p. 36, March 3, 1894, and Monatsh. f. prakt. Dermatologie, II., 1893. [4] Ed. Med. Journal, Feb. 1894, and Separatabdruck Balneologisch, Centralbl. II., 13. [5] Amer. J. Med. Sci., Feb., 1894, p. 191, and Bulletin Général de Thérapeutique. [6] Med. Record, Jan. 27, 1894, and Union Médicale, Dec. 30, 1893. [7] Amer. J. Med. Sci,. Nov., 1893, p. 597, and Münchener Med. Woch. [8] Amer. J. Med. Sci., Jan., 1894, p. 83. [9] Ibid. [10] Amer. J. Med. Sci., Feb., 1894, p. 192, and Centralblatt für Bakteriologie and Parasitenkunde. [11] Therapeutic Gazette, Feb. 15, 1894, p. 123, and Med. Record. [12] Les Nouveaux Remédes, Feb. 24, 1894, and Revue d'Hygiène. [13] Med. Record. Feb. 3, 1894, p. 160. [14] Brit. Med. Journal, Jan. 27, 1894, p. 175., [15] N. Y. Med. Record, March 3, 1894, p. 285. [16] Med. Record, Jan. 27, 1894, and Wiener Klinische Woch.

## Figures and Tables

**Figure f1:**